# TB Notification from Private Health Sector in Delhi, India: Challenges Encountered by Programme Personnel and Private Health Care Providers

**DOI:** 10.1155/2017/6346892

**Published:** 2017-08-06

**Authors:** Mahasweta Satpati, Sharath Burugina Nagaraja, Hemant Deepak Shewade, Prabhakaran Ottapura Aslesh, Blesson Samuel, Ashwani Khanna, Sarabjit Chadha

**Affiliations:** ^1^Population Services International (PSI), New Delhi, India; ^2^ESIC Medical College and PGIMSR, Bangalore, India; ^3^International Union Against Tuberculosis and Lung Disease (The Union), South-East Asia Regional Office, New Delhi, India; ^4^Department of Community Medicine, Academy of Medical Sciences, Pariyaram, Kerala, India; ^5^World Vision India, New Delhi, India; ^6^State TB Office, New Delhi, India

## Abstract

**Objective:**

To identify the challenges encountered by private health care providers (PHCP) to notify tuberculosis cases through a programme developed web-based portal mechanism called “NIKSHAY.”* Study Design*. It is a descriptive qualitative study conducted at two revised national tuberculosis control programme (RNTCP) districts of New Delhi. The study included in-depth interviews of PHCP registered with “NIKSHAY” and RNTCP programme personnel. Grounded theory was used to conceptualise the latent social patterns in implementation of tuberculosis case notification process and promptly identifying their challenges.

**Results:**

The analysis resulted in identification of three broad themes: (a) system implementation by RNTCP: it emphasizes the TB notification process by the RNTCP programme personnel; (b) challenges faced by PHCP for TB notification with five different subthemes; and (c) perceived gaps and suggestions: to improvise the TB notification process for the private health sector. The challenges encountered by PHCP were mainly related to unsystematic planning and suboptimal implementation by programme personnel at the state and district level. The PHCP lacked clarity on the need for TB notification.

**Conclusion:**

Implementation of TB notification among private health care providers requires systematic planning by the programme personnel. The process should be user-friendly with additional benefits to the patients.

## 1. Introduction

In India, tuberculosis continues to be the major public health problem. It accounts for 27% (2.8 million) of the estimated global annual incidence of 10.4 million tuberculosis (TB) cases [1]. Inappropriate diagnosis and irrational, incomplete treatment have led to emergence of drug resistant TB cases which are estimated to be 99,000 cases annually in the country [1, 2]. Globally, one-third of the nearly nine million people are estimated to fall ill with TB each year and many out of them could not be reached by TB programme. This “missing” patient, of population over three million, has stubbornly remained unchanged since 2007 [3].

During the year 2012, the Government of India had made notification of TB mandatory for the public and private health sector [4]. In 2014, global reporting system created by WHO measured a marked increase in global TB notifications. The annual total of new TB cases increased globally to 6%, mostly due to a 29% increase in TB notifications in India [5]. The increase can be attributed to the increased TB notification which otherwise was not notified earlier to the programme.

The RNTCP is promoting and advocating a web-based online reporting mechanism called “NIKSHAY” for TB notification. To start notifying the disease the practitioner had to get registered in the “NIKSHAY” web-based system. Initially, this process of registration into the system was facilitated by the RNTCP programme personnel.

The public health sector had begun notifying the disease through “NIKSHAY”: however, the notification from the private health sector remained suboptimal [1]. It is important considering more than 40% of TB cases are catered by private sector and nearly half of them are missed by the TB notification system [6]. In 2013, of all the total notified TB cases from New Delhi state no cases were notified by the private sector [7].

Due to expanding horizons and strategies under RNTCP, the programme managers and personnel were incapacitated by limited overburdened programme personnel [6, 8]. Conceptually, it was presumed that TB notification as a strategy in private health sector would be difficult to implement as there was no organized system in order to make notification a routine practice. There were only few studies on knowledge and gaps of private health care providers (PHCP) on TB notification, therefore, limiting information on the prevailing implementation issues related to notification system [8, 9]. Hence, we conducted this qualitative study to explore the challenges encountered by the RNTCP programme personnel and PHCP registered in “NIKSHAY” to routinely operationalize TB notification in New Delhi, India. The study finding has the potential to improve the manner in which the programme is implemented through PHCP at New Delhi, India.

## 2. Methods

### 2.1. Study Design

It is a qualitative study involving grounded theory approach. The data collection has been done through in-depth interviews for respondents selected through purposive sampling techniques. Content analysis was performed for data analysis.

### 2.2. Setting

The study was conducted at New Delhi during 1st December 2014 to 31st October 2015. A total of nine administrative districts are established and for the purpose of RNTCP programme implementation these are subdivided into 26 programme management units or RNTCP districts [10]. All the programme management units have similar human resources and logistics pattern for programme implementation. Based on the feasibility and convenience we purposively selected the following two RNTCP districts: New Delhi Municipal Corporation (NDMC) and the Guru Teg Bahadur (GTB) Chest clinic. These RNTCP districts have a population of 6.1 and 7.6 lakhs and had registered 1,417 and 1,914 TB patients, respectively, between October 2012 and September 2013 out of 1,52,181 TB cases registered at Delhi state [7]. The number of patients with TB notified by the PHCP in these two RNTCP districts during 2014 is shown in Table 1.

### 2.3. Study Participants and Sampling

A purposive sampling method with maximum variation was adopted depending on the respondents consent and availability. The aim of using a maximum variation in purposive sampling method was to purposively select heterogeneous population which maximize the diversity relevant for the research study. The RNTCP programme personnel and the PHCP registered in “NIKSHAY” between January 2014 and April 2015 at these two RNTCP districts constituted the study population. A total of 11 programme personnel and 19 PHCP were the respondents for in-depth interviews. The mean duration of time for the entire interviews was up to thirty minutes. The RNTCP programme personnel includes the District TB officer (DTO), who is overall in-charge for RNTCP implementation in the entire district, senior treatment supervisor (STS) who is responsible for TB treatment registration and supervision of patients with TB in 5,00,000 population, and the TB health visitor (TBHV) who is responsible for facilitating initiation of domiciliary treatment for patients with TB in 100,000 population.

The list of PHCP registered with “NIKSHAY” were obtained from the programme and for the purpose of sampling, the list of registered PHCP was further stratified into (a) laboratories, (b) private practitioners clinics/single private practitioners, and (c) hospitals/clinics/nursing homes. It was mainly done to obtain uniform representativeness from different type of health care providers. As it was a qualitative study the minimum numbers of sample size and sampling units were not required [11, 12]. Each of the RNTCP programme personnel cadre and the stratified PHCP were considered as sampling units [10].

### 2.4. Data Collection

Interview guides were prepared to elicit relevant information from RNTCP programme personnel and PHCP. Broadly, the programme personnel interview guide focused on the registration process of health facilities under RNTCP, their experiences in learning the phases of registration, and the web-based notification process. Emphasis was placed on challenges encountered for implementation and their plausible solutions [13]. Before interviewing the guides were pilot tested between January and June 2015. Informed consent was obtained from the respondents after explaining the purpose of the study and permission was sought to use audio recording during the interviews. Places for interviews were chosen by the respondents and interviews were conducted at a time which was convenient and comfortable for them. There were no repeat interviews and only the principal investigator (PI) engaged the respondents for interviews. The summary of the interviews were discussed with the respondents to ensure participant validation.

The PI stopped interviewing respondents once redundancy or consistency in responses was identified. The redundancy was defined as repetition of description of incidents for implementation of TB case notification process and its challenges.

### 2.5. Data Analysis and Management

To maintain consistency in data collection and in order to obtain respondent's concerns on TB notification, the grounded theory of data collection was adopted [13, 14]. The theory helped us to conceptualise the latent social patterns involved in implementation of TB case notification process and challenges. Interviews were transcribed and a constant comparative analysis was employed and an inductive analysis was carried out with focus on content to elicit codes and themes. Open type of coding method was employed, for the data analysis [15]. The analysis followed emergent codes from the responses of the individual respondents. To elicit codes the PI read and identified underlying meaning of the sentences and wrote them on the margins by clustering similar topics, unique topics, and other left over topics. The process was repeated till new codes emerged from the text [11]. After completing the process the PI identified themes for analysis [15]. Data relevant to each category was identified and examined using a process called constant comparison to establish analytical categories. The analysis of transcribed data was first performed by the PI and then independently analysed by the coinvestigators. For a common understanding of codes and establishing the intercoder reliability codes were discussed among two coinvestigators. Consultation within team was also held to decide about the next steps of data collection and to ensure the analysis followed the constant comparative steps of grounded theory to reach a saturation of details. To ensure descriptive validity and consistency both the researchers interpreted the data and made inferences about the findings [16]. The software Atlas Ti (version 6.0) was used for management of the data [17].

Complete, deidentified representative statements were included in the results to illustrate the main themes. The available themes from the programme personnel and PHCP interviews were conceptualised into broad categories, such as (a) system implementation by RNTCP; (b) challenges encountered by PHCP; (c) gaps and suggestions. A COREQ guideline was followed for reporting the qualitative aspects of the study [18].

### 2.6. Ethical Approval

Ethics Advisory Group, International Union Against Tuberculosis and Lung Disease (The Union), Paris, and Research Ethics Board of Population Services International approved this study. Administrative approval to conduct the study was obtained from RNTCP, Delhi, India. At times, when the programme personnel and private health providers were hesitant to provide written informed consent before speaking on sensitive issue such as “TB notification,” we sought oral consent for the interviews. The Ethics Committees approved the consent procedure and recording of interviews.

## 3. Results

**Figure 1:**
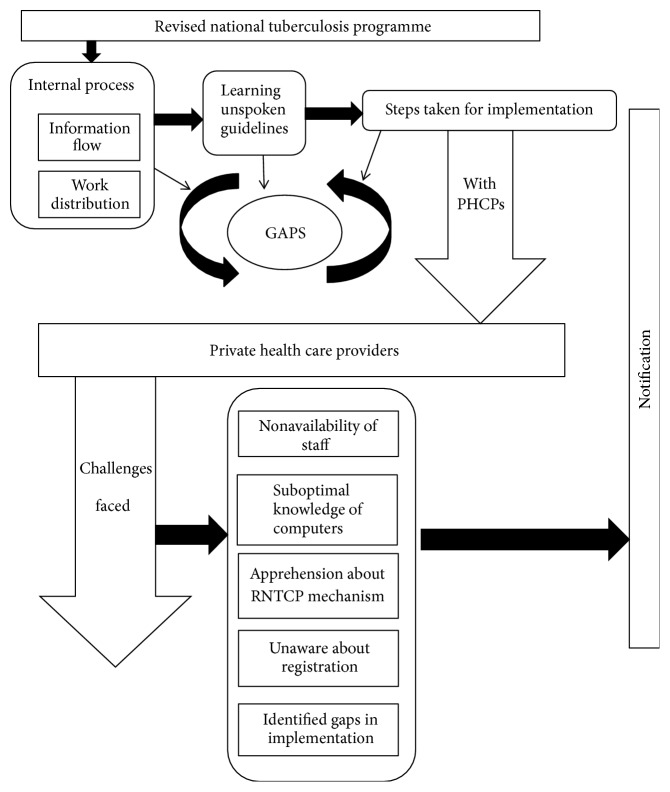
Theoretical model showing interrelationships of themes.

The complete process of how the activities were performed by RNTCP staff, flow of information, implementation of TB notification system and challenges faced by RNTCP and PHCP, gaps, and suggestions are discussed in this section. A theoretical model is represented showing interrelationships between themes (Figure 1()). The themes have been organized into the building blocks of the emerging theoretical model. The relationships between the themes are visualized with arrows to show the direction in which the process proceeded. The themes and information areas are labeled under RNTCP and PHCP. The details of the thematic structure observed and analysed during the interviews are enumerated below with detailed select quotes exemplifying the content.

### 3.1. System Implementation by RNTCP

The implementation process for TB notification system was driven by the state RNTCP. The strategy involved introduction of “NIKSHAY” web site at state and district levels. As a process the line lists of PHCP were prepared at district level and they were to be motivated for registering into the newer reporting mechanism “NIKSHAY.”

At first we received a mail from STO to DTO, and then we were informed. Then we informed tuberculosis health visitors that the private practitioner who does treatment of TB patients should be met, they met them took their registered number, contact number and prepared a list and submitted to office. (41 years, male, RNTCP programme personnel, Delhi)

Initially there was a difference as the processes were not planned. It took a one whole year to process the discussion. (50 years, male, RNTCP programme personnel, Delhi)

We were not informed that we have to enter about TB notification in the ‘NIKSHAY' web site. (39 years, male, RNTCP programme personnel, Delhi)

It was noticed that, at both of these RNTCP districts, there was a difference in the process of line list preparation, information that has to be disseminated to PHCP for TB notification, and registration in “NIKSHAY.”

At GTB RNTCP district the PHCP were provided with two options for notification: (i) to directly notify TB in the website or (ii) to submit the filled form and later submitting to programme personnel for further data entry and onward submission. while, at the NDMC RNTCP district, the field staff had informed PHCP to notify patients with TB treated by them and no specific process that has to be followed for notification was briefed to them.

We [field staff] did line list and in next visit we took DTO for field visit and submitted the line list for registration. (24 years, male, RNTCP programme personnel, Delhi)

We [field staff] had got line list provided by chest clinic which had Dr. Name alone in the list. We went there with one- one copy of format [notification format hard copy] and had told to private clinic that whatever TB patients come, about them you need to notify. (27 years, male, RNTCP programme personnel, Delhi)

We had given them two options that you can directly do in the computer in the ID we had created. The second option was we had asked them to keep register where they can record and give us a list. (41 years, male, RNTCP programme personnel, Delhi)

We informed them [PHCP] that if you are treating TB patients you have to inform us. If you face any problem you have to inform us. (41 years, male, RNTCP programme personnel, Delhi)

Programme personnel had informed that support was required for better implementation of TB notification system. The field staff required support for microplanning of field activities, training to carry the activities, and support of higher officials in interaction with PHCP.

We should know beforehand what we have to do. Some training should be there in which we will learn what we have to speak? We also need some ID card. Nobody accepts if you don't have any identity card. People ask from where you come. (39 years, male, RNTCP programme personnel, Delhi)

Someone needs to take the responsibility to speak to private Doctors, rest we can handle. People with higher position with higher responsibility can take initiative, as they have recognition in health as they have telephone number and cards. (25 years, male, RNTCP programme personnel, Delhi)

### 3.2. Challenges Faced by PHCP

**Figure 2:**
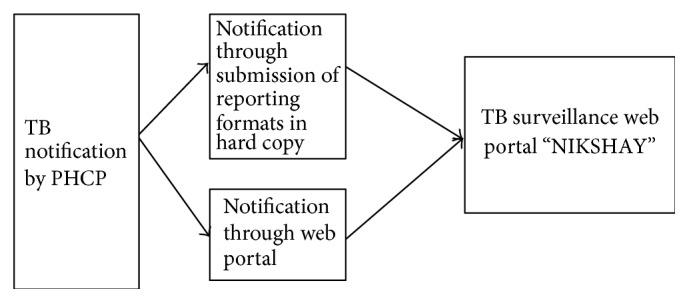
Schematic representation of TB notification process by PHCP in RNTCP system through “NIKSHAY” web portal. RNTCP: revised national tuberculosis control programme; NIKSHAY: web-based portal for notification and follow-up of patients with TB.

The notification process for PHCP involved notification of TB patient by submitting the hard copy formats to the government TB clinics or through direct notifying at web portal. The information through hard copies is filled by RNTCP staff and punched into the web portal. This information as either direct reporting or indirect reporting by PHCP goes to the TB notification web portal “NIKSHAY.” These two submission processes enable the private health sector to enter the patients with TB details into the website (Figure 2()). Themes identified as challenges for PHCP were as follows.

#### 3.2.1. Incomplete Patients Address

PHCP stated that there were gaps in recording TB patient details as many patients with TB did not remember their exact postal house address, few were migrants with temporary house address, and few had come from unauthorized colonies with no house address. Hence, incomplete house details were not considered valid for getting notified and thus remained unnotified.

Whoever patients come to us we try to get address from patients and they write from their hand. Still many illiterate people comes to us who do not know to write their address, few don't know about their address, some know their address of their lane but does not know home number. Many of the patients come from unauthorized colonies where addresses are not proper. Even in birth and death certificate government has tried to enforce the address still you may find the addresses are not correct. (52 years, male, private health care provider, Delhi)

#### 3.2.2. Nonavailability of Staff

Quite often, the private health facility encountered shortage of human resources that led to poor maintenance of records and registers at the facility. PHCP emphasized that they had no manpower to fill the individual forms and to maintain records.

I don't have any paramedical staff to do the work [notification]. On behalf of management I have said I will give phone numbers, other particulars like fathers name and address of patient will be given. It is being one year the notification process has started. And these cards will go back to our trust and will get destroyed in few months. (65 years, female, private health care provider, Delhi)

#### 3.2.3. Suboptimal Knowledge of Computers

Motivation to notify was also determined by challenges faced by PHCP at their individual levels. Lack of time during their routine practice and relatively less knowledge to deal with computers demotivated them to continue notification.

I being a general practitioner I am not very well versed with computer. To do notification in computer I have to ask my son to do for me. (46 years, male, private health care provider, Delhi)

I don't know much in computers as we are from earlier generation. People have not even given training on how to feed the data, once and twice they have informed us. There is a password and user identity, which we forget. (52 years, male, private health care provider, Delhi)

#### 3.2.4. Apprehension about RNTCP Mechanism

The prime source of earnings to the private health sector is patient's footfall to their health centers. Sharing details of the same patients to RNTCP gave them insecurity and a sense of loss of patients and monetary loss. They were also unhappy because they perceived that government might scrutinize their efficiency on diagnosis and treatment. They felt discomfort with a situation to disclose their charges to the patient for the services offered and were ignorant about the benefits rendered to the notified patients.

If I am treating, after curing then why should I send the information to those people [RNTCP]? I have notified but I don't know what benefit happened to me or patients. I don't know why we are doing it? If there is no monetary benefit why people should do notification? (47 years, male, private health care provider, Delhi)

There may be some apprehension in their mind (other PHCPs) that if they report about their patient directly then RNTCP will continue the treatment [DOTS] and the patient will go out of their hand. (65 years, female, private health care provider, Delhi)

#### 3.2.5. Unaware about Registration

Though the private health care providers were registered in “NIKSHAY” it was found that many of them were not aware about their name getting registered in the web site for treating TB patients.

I don't even know when they have registered me. I have no connections with them. They had come to meet us long back and have never come back. (32 years, female, private health care provider, Delhi)

I have no idea how come my name has come in your registration sheet we don't refer any TB patient. We do diagnostic for diabetics. (27 years, female, private health care provider, Delhi)

None of them had come to me. I would be very much interested to know more about it. (45 years, female, private health care provider, Delhi)

### 3.3. Perceived Gaps and Suggestions

Gaps were identified by different level of personnel involved in implementation of TB notification. The identified gaps by different personnel were related to their role and responsibility for TB notification. Gaps related to TB notification information punching in “NIKSHAY” were also discussed (Table 2). There were some perceived gaps stated by PHCP which were related to the implementation process conducted by RNTCP staff (Table 3). The private health sector also opined for better implementation of TB notification; most of these suggestions were related to training and conducting meetings and workshop for dissemination of information on notification among private health sector (Table 4).

## 4. Discussion

The TB notification in high-incidence countries is limited. A study based on literature search on notification system across high-incidence countries provided insights in private provider perceptions, including barriers to notification. Low notification rate was found in Republic of Korea, Pakistan, and Nigeria which was due to low knowledge associated with poor engagement of private providers on TB; lack of time was the major reason for not notifying while other concerns were confidentiality and poor knowledge of the reporting procedure. India is one among the few high-incidence countries, implementing electronic notification system recording details of TB patients [19].

In India, we conducted a qualitative study on TB notification which describes the challenges encountered by RNTCP programme personnel and PHCP. Our study findings revealed the operational issues in implementation of notification at various levels among PHCP starting from registration of PHCP in the notification system, the actual process of notification, and the perceptions associated with it among PHCP. The studies conducted elsewhere had evolved similar themes such as lack of complete knowledge about TB notification, lack of simplified operational mechanism of notification, and lack of trust and coordination with the government health system [9, 10, 20].

For implementation of TB notification by programme personnel at private health sector, the challenges faced by them were mainly the notification processes related issues and the differential strategies adopted by the programme at state and district levels. There appeared to be lack of microplanning at state and district level in terms of quantification of practitioners, planning, and implementation of systematic approach for involvement of practitioners and further mechanisms for regular follow-up of the involved practitioner. The component of TB notification for the programme at district level was perceived as another adjunct activity to the already existing RNTCP initiatives like TB-HIV (human immunodeficiency virus), drug resistance-TB, and TB-diabetes mellitus.

The programme had not prioritized the advocacy on the importance of TB notification upfront to providers and various myths prevailed among the private health care providers about TB notification [21]. There was also an apprehension among PHCP of delinking their patients from their services and sending them to RNTCP; the same was observed in a study conducted at Pune, Maharashtra [10]. The interaction with PHCP suggested that the bridge of faith and trust between RNTCP and PHCP was fragile and weak and it required attention to maintain transparency and developing dependency at both the sides. The PHCP had not experienced any gains either individually or to their patients upon notification [22].

Due emphasis of the programme should be concentrated on convincing the practitioners of the importance of TB notification [9, 23]. Awareness needs to be generated among the community for making the TB patients notified. This activity has to be widely publicized and supported through the involvement of community, NGOs, and social media. Innovative ideas need to be explored to make the tool user-friendly as the PHCP lacks clarity on functioning and usage of mobile phones and computers [23].

The gaps in the process of TB notification and suggestions to improvise the process as perceived by the programme personnel and the PHCP are enumerated in the tables. Though gaps are perceived at all levels by the programme personnel and PHCP, there are opinions for improvisation for notification. Accordingly, there is a need for the programme to popularise “NIKSHAY” as a notification tool. An advocacy forum of PHCP may be considered to motivate and streamline the notification among PHCP. The programme managers especially the district TB officers should judiciously take the initiative in meeting the PHCP by conducting meetings or workshops; thereafter, TBHV/STS should then follow up the PHCP at regular intervals. The programme personnel should also be adequately trained in the process with adequate transport facilities. It may be appropriate for the programme to collaborate with Indian Medical Association (IMA) which is the largest medical organization in the country to promote notification [23]. There is no universal successful formula to engage private health care providers; globally, there remains gaps in policy and practice for TB notification among the high burden TB countries [19].

### 4.1. Strength and Limitation of the Study

The strength of the study is that it was conducted under programmatic settings and reflected the ground reality. It represented the gaps and reasons behind poor notification, the detailed implementation process of TB notification, and strategies and challenges faced by PHCP. The limitations of the study included the following: (a) involvement of only two RNTCP districts of Delhi state may not reflect the challenges across the country and, hence, should be interpreted with caution; (b) although data saturation was achieved the additional themes might have emerged if additional nonregistered PHCP in “NIKSHAY” would have been interviewed to understand why other providers are not getting registered in “NIKSHAY” and interested to notify.

To conclude, the challenges in TB notification vary across the RNTCP personnel and PHCP. Notification of TB disease is an important monitoring and surveillance activity and the programme has to nurture its needs efficiently and establish a supportive environment for notification, while it is prudent that the importance and value of this tool are lost if not properly implemented. Implementation of TB notification among private health care providers requires systematic planning by the programme personnel and the process should be user-friendly with additional benefits to the patients and health care providers.

## Figures and Tables

**Table 1:** Notification profile of “NIKSHAY” registered PHCP in NDMC and GTB RNTCP districts of Delhi state, 2014.

Stratification of private health care providers	NDMC RNTCP district			GTB RNTCP district		
	Number registered in NIKSHAY	Number notifying patients with TB	Number of patients with TB notified	Number registered in NIKSHAY	Number notifying patients with TB	Number of patients with TB notified
Laboratories	2	2	**178**	16	0	**0**
Private practitioners clinic/single private practitioners	27	0	**0**	21	1	**90**
Hospitals/clinics/nursing home	3	0	**0**	5	3	**0**

Total	32	2	**178**	42	4	**90**

RNTCP: revised national tuberculosis control programme; NIKSHAY: web-based portal for notification and follow-up of patients with TB; NDMC: New Delhi Municipal Corporation; GTB: Guru Teg Bahadur Chest clinic.

**Table 2:** Gaps identified by RNTCP Personnel during the process of PHCP “NIKSHAY” registration and continued notification at two RNTCP districts of Delhi state, 2105.

District TB officer	Delayed start and the process does not take place properly
	Lack of strategy planning
	No monthly or quarterly targets

STS/STLS	Burdened higher officials
	Lack of rapport building with private practitioners
	TBHV, STS/STLS have poor recognition among private practitioners

TBHV	Vast areas to cover and short time for reporting information
	No transportation facilities
	No formal training on process

RNTCP: revised national tuberculosis control programme; NIKSHAY: web-based portal for notification and follow-up of patients with TB.

**Table 3:** Gaps perceived by RNTCP personnel and PHCP for smooth implementation of TB notification, at two RNTCP districts of Delhi state, 2015.

District TB officer	Double entries of the same TB cases at different places
	More chances of getting wrong information from field and wrong entries
	Difficulty in identification of entered address

Management information system personnel	Confusion with the process and reasons for data entry
	No clear information on indicators and which details to go where
	No guidance from superiors and miss communication on indicators

Private health care provider	No follow-up from RNTCP staff
	No proper information on the reasons of TB notification
	No perceived benefits are stated by RNTCP
	No trainings or meetings or communication materials given
	Confusion on intervention earlier done on public private mix strategy and TB notification

RNTCP: revised national tuberculosis control programme; NIKSHAY: web-based portal for notification and follow-up of patients with TB.

**Table 4:** Suggestions for smooth implementation of TB notification among “NIKSHAY” registered PHCP at two RNTCP districts of Delhi state, 2015.

Respondents	Suggestions
RNTCP personnel	
TBHV	Conduct prior meeting or workshop to give information on “NIKSHAY” and TB notification to TBHVs
	DTO should take steps and inform his staffs to collect data for 15 days or once in a month from private health care providers
	Conducting meeting with private health care providers on TB notification
STS/STLS	Government should provide a notice about TB notification to private practitioners and should be made a rule
	Indian Medical Association (IMA) at national and district level should inform all the doctors about this and should be sensitized on this
	Before making guideline at policy level ground reality should be understood
DTO	If notified once they should be known to others also in other district or state
	DTO should visit to meet private practitioners and vehicle can be hired for full time; many things can happen from DTOs part
	Duplication of TB numbers for same person at different places should be stopped

PHCP	DOT centers should be provided to private health care providers
	Follow-up process from RNTCP will be an effective solution to improve notification
	Notification process should be made convenient and easy
	Group for qualified and nonqualified PHCP should be build and educate them from RNTCP

RNTCP: revised national tuberculosis control programme; NIKSHAY: web-based portal for notification and follow-up of patients with TB; STS: senior treatment supervisor; STLS: senior TB laboratory supervisor; DTO: district tuberculosis officer; TBHV: TB health visitor; PHCP: private health care providers.
